# Genomic Profile of Non-Small Cell Lung Cancer in a Spanish Cohort: A 2-Year Descriptive Study Using Next-Generation Sequencing

**DOI:** 10.3390/genes17020209

**Published:** 2026-02-09

**Authors:** Miguel Carnero-Gregorio, Enzo Perera-Gordo, Vanesa de la Peña-Castro, Antonio Fernández-Gómez, Carmen Rodríguez-Cerdeira

**Affiliations:** 1 Department of Pathology, Hospital Universitario de Gran Canaria Dr. Negrín, 35010 Las Palmas de Gran Canaria, Spain; mcargre@gobiernodecanarias.org (M.C.-G.); epergor@gobiernodecanarias.org (E.P.-G.); afergomd@gobiernodecanarias.org (A.F.-G.); 2 Fundacion Vithas, Grupo Hospitalario Vithas, Principe de Vergara 109, 28002 Madrid, Spain; 3 Fundación Canaria Instituto de Investigación Sanitaria de Canarias, 35012 Las Palmas de Gran Canaria, Spain; nesa206@gmail.com; 4 Dermatology Department, Grupo Hospitalario (CMQ Concheiro), Manuel Olivie 11, 36203 Vigo, Spain; 5 Department of Health Sciences, Campus of Vigo, University of Vigo, As Lagoas, 36310 Vigo, Spain; 6 European Women’s Dermatologic and Venereologic Society (EWDVS), 36700 Tui, Spain

**Keywords:** non-small cell lung cancer, next-generation sequencing, genomic profiling, targeted therapy, *KRAS*, *EGFR*, *ALK*, gene fusions, co-mutations

## Abstract

Background/Objectives: Next-generation sequencing (NGS) has become the standard of care for identifying actionable genomic alterations in non-small cell lung cancer (NSCLC). This study aims to describe the clinicopathological characteristics and genomic landscape of a non-selected cohort of NSCLC patients from the Canary Islands (Spain), analyzed during the first two years of our Molecular Diagnosis Unit’s operation. Methods: We conducted an observational, retrospective study including 448 tumors from 446 patients diagnosed between March 2023 and March 2025. Genomic profiling was performed using amplicon-based NGS panels (Oncomine™ Focus and Precision Assays) on semiconductor sequencing platforms to detect single-nucleotide variants (SNVs), indels, copy number alterations (CNAs), and gene fusions from DNA and RNA. Results: Actionable alterations were identified in 55.1% of tumors. The most prevalent alterations were found in *TP53* (29.5%), *KRAS* (27.2%), and *EGFR* (14.1%), with *KRAS* G12C being the most frequent variant. Stratified analysis revealed a high prevalence of *ALK* fusions in patients < 50 years (33.3%). Crucially, and in stark contrast with traditional exclusion criteria, 54.0% of *EGFR* mutations and 50.0% of *ALK* fusions were detected in patients with a history of smoking. Concomitant alterations were observed in 34.8% of cases, with *TP53* being the most common co-mutation partner. Conclusions: Our real-world data confirm the feasibility and clinical value of routine NGS testing for NSCLC. The findings highlight specific genomic patterns in this population and demonstrate that smoking status should not preclude comprehensive molecular testing for canonical drivers.

## 1. Introduction

Lung cancer constitutes the leading cause of cancer death worldwide, with an estimated 1.8 million deaths and 2.2 million new cases registered in 2020 [1,2,3,4]. Approximately 85% of all lung cancer cases correspond to non-small cell lung cancer (NSCLC) [3,4,5], with adenocarcinoma and squamous cell carcinoma being the most common histological types [1]. A major clinical difficulty lies in the fact that a high percentage of patients, around 60–65%, are diagnosed at locally advanced or metastatic stages [4,5], significantly limiting curative treatment options.

In recent decades, the management and therapeutic approach to NSCLC have undergone a radical transformation, evolving from a unique approach based on cytotoxic chemotherapy towards a precision medicine model guided by molecular biomarkers [2,6,7]. This strategy is based on the identification of specific genomic alterations, known as oncogenic drivers, which drive tumor growth and can be selectively inhibited [8]. This has allowed for the identification of patient subgroups that benefit from targeted therapies, significantly improving survival [2,6]. The identification of actionable biomarkers has become essential to guide therapeutic decisions, enabling more effective and less toxic treatments.

National and international clinical guidelines, such as those of the Spanish Society of Pathology (SEAP) and the Spanish Society of Medical Oncology (SEOM), the National Comprehensive Cancer Network (NCCN), or the European Society for Medical Oncology (ESMO), have rapidly expanded the list of predictive biomarkers whose determination is considered essential for therapeutic decision-making in NSCLC. Thus, key molecular biomarkers that must be mandatorily determined in patients with NSCLC include mutations in *EGFR*, *BRAF*, *KRAS*, and *MET*, as well as rearrangements or fusions in *ALK*, *ROS1*, *NTRK*, and *RET*, in addition to PD-L1 expression, which is a crucial biomarker for immunotherapy [1,9,10,11]. This essential panel includes genomic alterations with approved therapies. Among them are *KRAS* mutations, which are the most frequent, present in 25–30% [11,12,13]; activating mutations of the *EGFR* gene, present in 10–20% of the Caucasian population and up to 50% of the Asian population [2,3,14]; *BRAF* V600E mutations (2–4%) [2,3]; splicing mutations causing *MET* exon 14 skipping (3–4%) [2,13,15,16]; and rearrangements of the *ALK* (2–7%), *ROS1* (1–2%), *RET* (1–2%), and *NTRK* (0.1–1%) genes [2,11,17,18,19].

Furthermore, the field of precision oncology continues to expand with the consolidation of emerging biomarkers. Mutations in *ERBB2* (*HER2*), mainly exon 20 insertions occurring in 2–4% of adenocarcinomas [3,11], have become actionable thanks to new drugs such as antibody–drug conjugates [2,20,21,22]. Along with these, the identification of concomitant mutations in genes such as *TP53*, *STK11*, or *KEAP1* has an increasingly relevant prognostic and predictive value, influencing the response to targeted therapies and immunotherapy [10,13,23,24,25]; for example, the coexistence of a *TP53* mutation with a main driver (e.g., *EGFR* or *ALK*) is consistently associated with a worse prognosis and shorter duration of response to targeted therapy [26].

The growing number of clinically relevant biomarkers has surpassed the capabilities of traditional single-gene testing methods, such as real-time polymerase chain reaction (RT-PCR) or fluorescence in situ hybridization (FISH), making sequential gene-by-gene analysis strategies inefficient, as they consume a large amount of tissue—often scarce—and delay the acquisition of a complete molecular profile [2,8,23,27,28]. In this context, next-generation sequencing (NGS) has consolidated itself as the standard of care recommended by major scientific societies [1,2,3,8,9,10,11]. NGS allows for the simultaneous analysis of multiple genes and types of alterations—including single-nucleotide variants (SNVs), small insertions and deletions (indels), copy number alterations (CNAs), and gene fusions—from a single sample, being more cost-effective, saving time, and maximizing the detection of actionable alterations [23,27,28,29,30,31]. This capability for comprehensive molecular profiling is crucial, especially because NSCLC is a molecularly heterogeneous disease where the coexistence of multiple alterations, or the presence of rare and uncommon mutations, can influence treatment response and resistance.

For comprehensive genomic characterization, an approach combining DNA and RNA analysis is considered ideal [10]. RNA-based NGS has proven to be superior and more sensitive for the detection of gene fusions (*ALK*, *ROS1*, *RET*, *NTRK*), whose breakpoints may be located in large introns that hinder their detection by DNA NGS, and for splicing events, such as *MET*ex14 [6,10,13,15,16,17,18,32,33]. The implementation of an NGS-based workflow in routine clinical practice is, therefore, a fundamental pillar of modern thoracic oncology.

This study aims to describe the experience of the first two years of the Molecular Diagnosis Unit at the Hospital Universitario de Gran Canaria Dr. Negrín (HUGCDN) in sequencing a non-selected cohort of patients with NSCLC, detailing the clinicopathological characteristics and the spectrum of genomic variants found using NGS panels.

## 2. Materials and Methods

### 2.1. Study Design and Population

An observational and retrospective study was conducted, including tumor samples from all patients diagnosed with NSCLC at the Pathology Department of HUGCDN between 1 March 2023 and 31 March 2025. All NSCLC cases were included in the molecular analysis, regardless of histological type or tumor stage. The cohort represents a consecutive series of patients referred for molecular testing according to institutional protocols, with no age-based exclusion criteria applied for this study. Patient consent was waived due to the retrospective nature of the study, which involved the analysis of anonymized clinical data derived from routine care, as authorized by the Ethics Committee.

Out of a total of 466 patients diagnosed in the period, 446 were included in the sequencing study. Twenty cases (4.3%) were excluded due to insufficient quantity or quality of the extracted genetic material or due to the patient’s death prior to molecular analysis. Demographic and clinicopathological data, including age, sex, histological type, and tumor stage (classified according to the 8th edition of the AJCC TNM staging manual [American Joint Committee on Cancer, Chicago, IL, USA]), were collected from electronic medical records.

### 2.2. NGS Analysis

Molecular analysis was performed at the Molecular Diagnosis Unit of the Pathology Department. The technological workflow was modified during the study period.

- Period 1 (1 March 2023–31 December 2023): The commercial Oncomine^TM^ Focus Assay panel (OFA, Thermo Fisher Scientific, Waltham, MA, USA) [34] was used, and sequencing was performed on a GeneStudio^TM^ S5 System (Thermo Fisher Scientific, Waltham, MA, USA).
- Period 2 (1 January 2024–31 March 2025): The Oncomine^TM^ Precision Assay panel (OPA, Thermo Fisher Scientific, Waltham, MA, USA) [35] was implemented on the automated Genexus^TM^ Integrated Sequencer (Thermo Fisher Scientific, Waltham, MA, USA).

Both the GeneStudio^TM^ S5 System and Genexus^TM^ Integrated Sequencer employ semiconductor sequencing systems, where nucleotide incorporation detection during synthesis occurs via pH changes generated by hydrogen ion release, differentiating them from traditional optical systems. Both assays are multigene panels based on Ion AmpliSeq^TM^ technology (Thermo Fisher Scientific, Waltham, MA, USA), allowing simultaneous detection of SNVs, indels, CNAs, and gene fusions from DNA and RNA extracted from formalin-fixed paraffin-embedded (FFPE) samples. Technical differences and gene coverage between both panels are detailed in Table 1.

### 2.3. Statistical Analysis

A descriptive analysis of variables was performed by sex, age group, tumor histological type, tumor stage, and smoking status. For age stratification, the cohort was divided into three groups: <50 years, 50–69 years, and ≥70 years. Data are presented as absolute frequencies and percentages.

## 3. Results

### 3.1. Demographic and Clinicopathological Characteristics of the Cohort

During the study period, a total of 448 NSCLC tumors corresponding to 446 patients were analyzed. Two patients presented with multiple primary tumors (one case with two squamous cell carcinomas and another with an adenocarcinoma and a squamous cell carcinoma); therefore, the description of histological and molecular characteristics is based on the total of 448 tumors.

The demographic and clinicopathological characteristics of the cohort are summarized in Table 2. The median age at diagnosis was 67 years (range: 34–94 years). The cohort showed a predominance of male patients [*n* = 286, 64.1%] and an age group between 50 and 69 years [*n* = 259, 58.1%].

The most frequent histological type was adenocarcinoma [*n* = 267, 59.6%], followed by squamous cell carcinoma [*n* = 119, 26.6%]. Notably, 50.4% of tumors [*n* = 226] were diagnosed at stage IV. The complete distribution across other stages was stage IA [*n* = 53], stage IB [*n* = 28], stage IIA [*n* = 9], stage IIB [*n* = 34], stage IIIA [*n* = 37], stage IIIB [*n* = 31], and stage IIIC [*n* = 11]. Tumor staging data were unavailable for 19 patients at the time of data collection.

Regarding smoking status, the majority of patients are current smokers [*n* = 205, 45.9%], 148 have been smokers at some point in their lives, and 40 have never smoked. Smoking status is unknown for 53 patients (Table 2).

#### Correlation of Histology with Smoking Status

The distribution of histological types according to patient smoking status was analyzed (Table 3), with data available for 395 of the 448 tumors (88.2%). A strong association was observed between squamous cell carcinoma and smoking; 112 of the 113 cases (99.1%) with available data occurred in smokers (*n* = 62) or former smokers (*n* = 50), and only one case (0.9%) was detected in a non-smoking patient. Adenocarcinoma, although also more frequent in smokers (*n* = 117) and former smokers (*n* = 85), presented the highest proportion of cases in non-smoking patients (*n* = 37), representing 92.5% (37/40) of all tumors diagnosed in this group.

### 3.2. General Spectrum of Genomic Alterations

**Figure 1:**
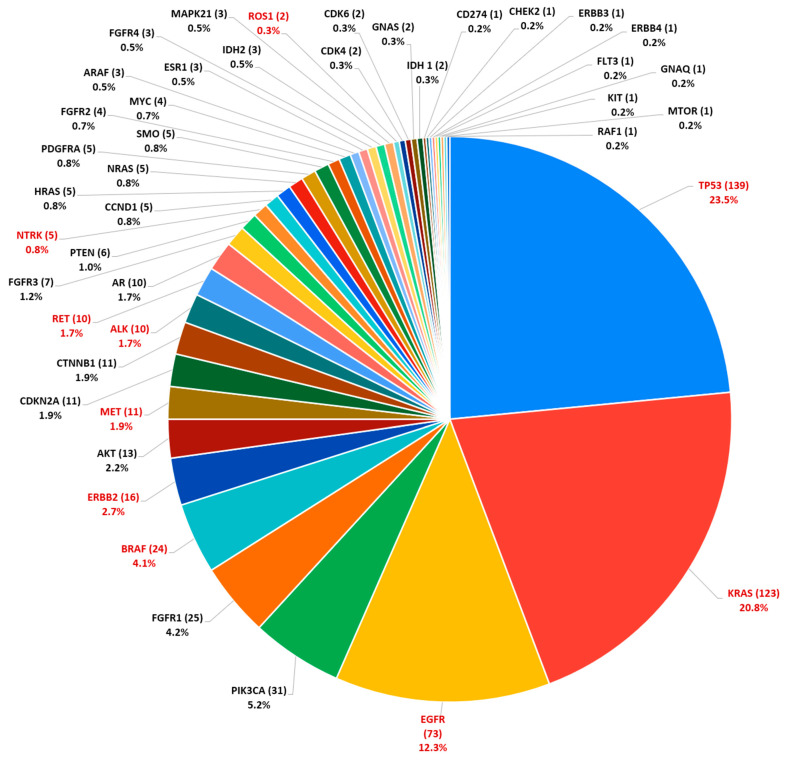
Number of alterations (in parentheses) and percentage of those found in genes included in the OPA and OFA panels in the 448 sequenced tumors.

In the set of 448 tumors analyzed by NGS, a total of 616 genomic alterations were identified, distributed across 43 genes. These alterations comprise a diverse range of molecular variants, including SNVs, CNAs, and gene fusions. The distribution and frequency of these alterations per gene are represented in Figure 1.

The most prevalent alterations were found in the *TP53* (*n* = 139) and *KRAS* (*n* = 123) genes, followed by *EGFR* (*n* = 73), *PIK3CA* (*n* = 31), and *FGFR1* (*n* = 25). It is important to note that the number of *TP53* alterations is likely underestimated, as this gene was not included in the OFA panel used during 2023, being analyzed only in samples processed from 2024 onwards with the OPA panel. Conversely, all alterations in the *ARAF* gene (*n* = 3) were detected in 2023, as this gene is not part of the OPA panel coverage.

### 3.3. Profile of Genomic Alterations in the NSCLC Cohort

A total of 395 genomic alterations were detected in genes with direct clinical relevance and potential therapeutic implications in NSCLC across the 448 analyzed tumors (*ALK*, *BRAF*, *EGFR*, *ERBB2*, *KRAS*, *MET*, *NTRK*, *RET*, *ROS1*, and *TP53*). The full spectrum and frequency of variants detected in the genes of interest are summarized in Table 4.

The most frequent genomic alterations occurred in the *TP53* gene, detected in 132 tumors (29.46%), followed by alterations in *KRAS*, detected in 122 tumors (27.23%), and in *EGFR*, present in 63 cases (14.06%). Alterations in *BRAF* were found in 24 tumors (5.36%), and in *ERBB2* in 16 tumors (3.57%). Alterations involving the *MET* gene were found in 11 cases (2.46%). Gene fusions were less common, with rearrangements in *ALK* and *RET* detected in 10 tumors each (2.23% for both), *NTRK* fusions in five tumors (1.12%), and *ROS1* fusions in two tumors (0.45%).

#### 3.3.1. Profile of Genomic Alterations in 10 Clinically Relevant Genes

Regarding the type of molecular alterations identified, details for 10 clinically relevant genes (*ALK*, *BRAF*, *EGFR*, *ERBB2*, *KRAS*, *MET*, *NTRK*, *RET*, *ROS1*, *TP53*) are as follows.

**Figure 2:**
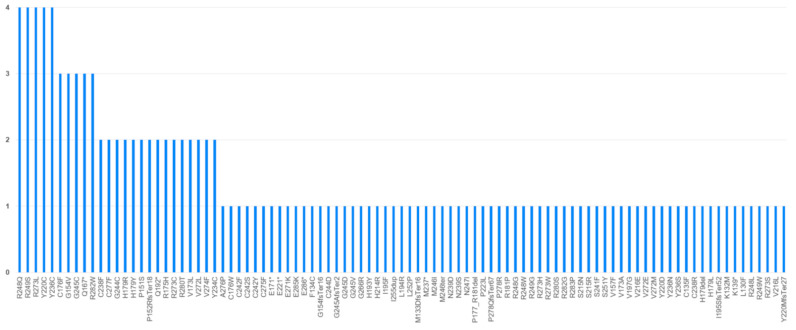
Distribution of variants found in the *TP53* gene (the four intronic variants are not shown).

- *EGFR*: A total of 73 alterations were identified, as some tumors presented more than one alteration in this gene. Analysis of SNVs and small indels revealed two alterations in exon 18 (indel E709-E710 [2.78%]), 26 deletions in exon 19 [35.62%], eight alterations in exon 20 (including four insertions [5.48%], two S768I variants [2.74%], and two V774M variants [2.74%]), 14 alterations in exon 21 (of which 12 were L858R variants [16.44%], one L861Q variant [1.37%], and one V843L variant [1.37%]), and one G1054W variant in exon 25 [1.37%]. Additionally, 22 CNAs were detected in this gene [30.14%], all of them gains. The results are shown in Table S1.
- *KRAS*: Of the 123 SNVs detected in *KRAS* (one tumor presented two concomitant *KRAS* alterations), the most common was G12C [*n* = 46, 37.40%], followed by G12V [*n* = 33, 26.83%] and G12A [*n* = 8, 6.50%]. Other recurrent variants included G12D [*n* = 7, 5.69%], G13D and G13C [*n* = 6, 4.88%], Q61H [*n* = 4, 3.25%], Q61L [*n* = 3, 2.44%], G12R [*n* = 2, 1.63%], and D30E, Q61R, A146T, G12S, and V8L [*n* = 1, 0.81%]. Additionally, two gains were detected [*n* = 2, 1.63%]. The complete distribution of *KRAS* variants is shown in Table S2.
- *BRAF*: A total of 24 alterations were found in our series [5.36%], of which one-third corresponded to the V600E variant [*n* = 8, 33.33%] and 14 were non-V600E variants (G469A [*n* = 3, 12.50%], G464V [*n* = 3, 12.50%], G596R [*n* = 3, 12.50%], D594N [*n* = 2, 8.33%], G466R [*n* = 2, 8.33%], G469R [*n* = 1, 4.17%], K601E [*n* = 1, 4.17%], and L597V [*n* = 1, 4.17%]). The results are shown in Table S3.
- *ALK*: A total of 10 alterations were detected in this gene, including six gene fusions (*EML*(13)::*ALK*(20) [*n* = 3, 30.0%] and *EML*(6)::*ALK*(20) [*n* = 3, 30.0%]), three SNVs (F1027L [*n* = 1, 10.0%], M1273T [*n* = 1, 10.0%], and L1187M [*n* = 1, 10.0%]), and one intronic variant [*n* = 1, 10.0%]. The distribution of alterations in *ALK* is shown in Table S4.
- *MET*: Of the 12 alterations identified [2.68%], nine corresponded to splicing events affecting exon 14 (*MET*ex14 skipping), with seven of them being variants found directly in the RNA analysis [58.33%] and two D1028N variants found in the DNA analysis [16.67%] (one of them concomitant with RNA alteration). Of the remaining three alterations, two corresponded to gain-type CNAs [16.67%] and one to the H1112Y variant [8.33%]. The results are shown in Table S5.
- *RET*: A total of 10 alterations [2.23%] of diverse nature were found in the 448 analyzed tumors: three gene fusions (*CCDC6*(1)::*RET*(12) [10.0%], *KIF5B*(15)::*RET*(12) [10.0%], and a 5′-3′ expression imbalance without an identified partner [10.0%]), six SNVs (R886Q [*n* = 3, 30.0%], R886W [*n* = 1, 10.0%], V804M [*n* = 1, 10.0%], and E768K [*n* = 1, 10.0%]), and one loss-type CNA [10.0%]. The distribution of these alterations is shown in Table S6.
- *NTRK*: A total of five alterations were identified in the 448 tumors [1.12%], including three fusions (*TPM3*(7)::*NTRK1*(10) [*n* = 1, 20.0%], *TPR*(21)::*NTRK1*(12) [*n* = 1, 20.0%], and *STRN*(3)::*NTRK2*(15) [*n* = 1, 20.0%]) and two gain-type CNAs [40.0%]. The results are shown in Table S7.
- *ROS1*: Two gene fusions [0.45%] were detected, with known fusion partners: *EZR*(10)::*ROS1*(34) [50.0%] and *CD74*(6)::*ROS1*(34) [50.0%]. The results are shown in Table S8.
- *ERBB2 (HER2)*: A total of 16 alterations were detected in the 448 tumors analyzed [3.57%], including five gain-type CNAs [31.25%], seven missense variants (D769H [*n* = 1, 6.25%], R683Q [*n* = 1, 6.25%], I370M [*n* = 1, 6.25%], G660D [*n* = 1, 6.25%], S310F [*n* = 1, 6.25%], and S310Y [*n* = 2, 12.50%]), and four insertions (Y772_A775dup) [25.0%]. The results of these findings are shown in Table S9.
- *TP53*: A total of 139 variants were detected in tumors sequenced in 2024 and 2025 with the OPA panel, as this gene was not present in the OFA panel used in 2023 and, therefore, could not be sequenced. Of the total 139 variants, four were found in intronic zones. The distribution of the remaining 135 variants is shown in Figure 2.

#### 3.3.2. Correlation of Genomic Alterations with Histological Type

As detailed in Table 3, the distribution of genomic alterations showed a strong association with tumor histological type. Alterations considered drivers of adenocarcinoma were predominantly found in this type; 100% of *ALK* fusions (10/10), 81.97% of *KRAS* mutations (100/122), and 81.82% of *MET* alterations (9/11) were detected in adenocarcinomas. Similarly, *BRAF* mutations (79.17%, 19/24) and *NTRK* (60.00%, 3/5) and *RET* (60.00%, 6/10) fusions were more prevalent in this histology.

*EGFR* mutations were also more frequent in adenocarcinomas [*n* = 46, 73.02%]; however, it is noteworthy that 14 *EGFR* mutations were identified in squamous cell carcinomas [22.22%]. In the NSCLC-NOS group, the most common alteration was the *KRAS* mutation [*n* = 17, 32.69%]. All alterations detected in *BRAF*, *EGFR*, *KRAS*, *MET*, *RET*, and *ERBB2* genes in squamous cell carcinoma were in patients ≥50 years old. The distribution of gene alterations across different tumor types is shown in Table 5.

#### 3.3.3. Correlation of Genomic Alterations with Tumor Stage

Most genomic alterations were identified in advanced-stage tumors (Table 6). A total of 58.20% of *KRAS* mutations [*n* = 71], 61.90% of *EGFR* mutations [*n* = 39], and 50.00% of *BRAF* mutations [*n* = 12] were detected in stage IV tumors. This trend was even more pronounced for gene fusions, where 80.00% of *ALK* rearrangements [*n* = 8] and 90.00% of *RET* rearrangements [*n* = 9] were found in metastatic disease. Conversely, mutations in *KRAS*, *EGFR*, and *BRAF* were also observed with notable frequency in early stages (I and II).

#### 3.3.4. Correlation of Genomic Alterations with Sex and Age Group

Stratified analysis by sex and age revealed distinctive distribution patterns (Table 7). *EGFR* mutations were notably more frequent in women than in men (39 vs. 24 cases), a difference accentuated in the ≥70 years age group (18 women vs. 10 men). Conversely, *KRAS* and *BRAF* mutations were more prevalent in men (75 vs. 47 cases for *KRAS*; 16 vs. 8 for *BRAF*).

Age distribution also showed significant trends. Gene fusions, particularly those of *ALK*, were exclusively found in the <70 years age group, where 10 cases (100%) were identified. In contrast, the majority of *KRAS* mutations [*n* = 81, 66.39%] were concentrated in the 50 to 69 years age group.

#### 3.3.5. Correlation of Genomic Alterations with Smoking Status

The distribution of the 10 main genomic alterations according to smoking status was analyzed (Table 8). A marked association was observed between smoking (smokers or former smokers) and mutations in *KRAS* (109/122, 89.3%) and *TP53* (107/132, 81.1%). Conversely, *EGFR* alterations showed a high prevalence in non-smoking patients (22/63, 34.9%), although it is noteworthy that the majority of *EGFR* alterations (34/63, 54.0%) were detected in patients with a history of smoking (smokers or former smokers). Alterations found in *ALK* also showed a significant presence in smoking patients (5/10, 50.0%).

Regarding the profile of variants found in these genes, it was observed that of the six *ALK* fusions, two were present in a smoker and three in a non-smoker; smoking data was unavailable for the other. V600E variants were found in four smokers, two former smokers, and two non-smokers. Non-V600 variants were only found in smokers or former smokers.

Regarding *EGFR*, the most frequent alterations in smokers and former smokers were amplifications [*n* = 17], followed by exon 19 deletions [*n* = 8] and the L858R variant [*n* = 5]; in non-smokers, the most frequent alteration was exon 19 deletion [*n* = 10], followed by the L858R variant [*n* = 5] and amplifications [*n* = 4].

In *KRAS*, the G12C variant was only found in one non-smoker [*n* = 1], with the rest found in smokers or former smokers [*n* = 27 and *n* = 16, respectively] and two in patients with unknown smoking status. Regarding the other five variants following in frequency (G12V, G12A, G12D, G13D, and G13C), their presence in smokers, former smokers, and non-smokers is summarized in Table 9, being more common in patients with a present or past smoking history.

Of the two cases of *ROS1* fusions, smoking information was available for neither. Regarding *MET*, of the seven cases of exon 14 skipping, six were present in smokers and former smokers [*n* = 3 for each subgroup] and one in a non-smoking patient. All *RET* fusions were found in patients with a history of smoking; *RET* fusions where the fusion partner was identified (*CCDC6* and *KIF5B*) were found in former smokers, while the fusion where the partner was unknown (imbalance) was found in a smoker. None of the missense variants nor the gains were found in non-smoking patients.

Of the two fusions found in *NTRK*, smoking information was unavailable (*STRN-NTRK2* and *TRP-NTRK1*), while the *TPM3-NTRK1* fusion was found in a non-smoking patient. Finally, in the *ERBB2* gene, only three Y772_A775dup alterations were found in non-smoking patients, while smoking history was unavailable for the other Y772_A775dup alteration. The rest of the alterations were found in smokers or former smokers.

#### 3.3.6. Concomitant Genomic Alterations

Co-occurrence analysis revealed a total of 172 co-mutations in the cohort. Of these, 86 corresponded to concomitant alterations between the 10 genes of main clinical interest (*EGFR*, *BRAF*, *KRAS*, *ALK*, *RET*, *MET*, *ROS1*, *NTRK*, *ERBB2*, and *TP53*). The most relevant patterns of concomitant alterations between two genes are detailed in Table 10.

The *TP53* gene was the most frequent co-alteration partner, confirming its role as the most common concurrent alteration in NSCLC. A high frequency of concomitant *TP53* alterations was observed with the two most prevalent alterations in the series: *KRAS* [*n* = 25, 5.58%] and *EGFR* [*n* = 11, 2.46%]. Furthermore, *TP53* was found in combination with other actionable drivers, including cases of *TP53* + *ALK* [*n* = 3, 0.67%], *TP53* + *BRAF* [*n* = 7, 1.56%], *TP53* + *MET* [*n* = 1, 0.22%], *TP53* + *HER2* [*n* = 3, 0.67%], *TP53* + *NTRK* [*n* = 1, 0.22%], and *TP53* + *TP53* [*n* = 3, 0.67%].

Beyond concomitant alterations with *TP53*, less frequent but clinically significant co-occurrences between oncogenic drivers were identified. These included two cases of *BRAF* with *EGFR* [0.45%] and four cases of *BRAF* with *KRAS* [0.89%], two cases of *KRAS* with *EGFR* [0.45%], and one case of *KRAS* with *RET* [0.22%]. Combinations of alterations in the *ERBB2* gene with *EGFR* [*n* = 2, 0.45%] and two cases of *EGFR* co-mutations [0.45%] were also detected.

Finally, high-complexity profiles were observed in some tumors, highlighting some cases of concurrent alterations in three genes (*ALK* + *KRAS* + *TP53* [*n* = 1, 0.22%], *BRAF* + *KRAS* + *TP53* [*n* = 2, 0.45%], *EGFR* + *MET* + *TP53* [*n* = 1, 0.22%], *EGFR* + *RET* + *TP53* [*n* = 1, 0.22%], and *EGFR* + *KRAS* + *TP53* + *TP53* [*n* = 1, 0.22%]).

## 4. Discussion

The implementation of NGS in our center has allowed for the characterization of the genomic landscape of NSCLC in the Canary Islands population. Our findings reveal a prevalence of actionable alterations (55.1%) consistent with other Western and Spanish cohorts, such as the ATLAS study [36], but with distinct local particularities. Beyond confirming the utility of NGS over sequential testing, our real-world data provide crucial insights into the complexity of co-mutation profiles and age-dependent genomic patterns, which have direct implications for patient management and prognosis.

For the analysis of our results, we stratified the cohort into three age groups (<50, 50–69, and ≥70 years). This division, although variable in the literature, is based on recurrent cut-off points that have demonstrated biological and clinical relevance [37]. Age significantly influences the molecular presentation of tumors, likely reflecting different exposures to carcinogens and the cellular aging process [38]. The definition of “young patient” often uses thresholds of 40 or 50 years, while the advanced age group is usually defined from 60 or 70 years onwards [37,39,40]. This stratification allows us to explore if our cohort replicates the age-dependent genomic patterns previously described.

Several large-scale studies have reported that younger patients present a higher frequency of gene fusions (*ALK*, *ROS1*, *RET*) and certain *EGFR* mutations, while older patients tend to show a higher prevalence of *KRAS* mutations, *MET*ex14 splicing alterations, and a higher tumor mutational burden (TMB) [37,39,40]. Therefore, analyzing our findings across these age groups will allow us to contextualize the molecular profile of our population and evaluate the distribution of actionable biomarkers across different age segments.

Our findings, obtained in a cohort of 448 tumors from predominantly Canarian patients, largely reflect the genomic landscape described in Caucasian populations, albeit with certain peculiarities deserving discussion compared to the literature [41,42]. The overall frequency of actionable alterations detected (55.1% of tumors with at least one variant in *KRAS*, *EGFR*, *BRAF*, *MET*, *ALK*, *RET*, *NTRK*, or *ROS1*) underscores the importance of implementing broad genomic profiles in our clinical practice.

Activating mutations in *EGFR* were detected in 14.06% of our tumors, a prevalence fitting perfectly within the 10–20% range described for Caucasian patients [11,43] and specific European (12.8–14.1%) and Spanish (14–14.5%) data [41,44]. However, the distribution of canonical variants showed a slight deviation: exon 19 deletions represented 35.62% of *EGFR* mutations and L858R 16.44%, whereas in the literature, they usually account for around 57% and 23%, respectively [45]. This lower relative proportion of L858R could be a particular characteristic of our population or due to sample size. Exon 20 insertions constituted 5.48% of *EGFR* mutations, within the expected range of 4–12% [44,46,47].

A notable finding was the detection of *EGFR* mutations in 14 squamous cell carcinomas (22.22% of total mutated *EGFR*). Of these alterations, three corresponded to deletions in exon 19 and one alteration to L858R. Although traditionally associated with adenocarcinoma, this result highlights the importance of not ruling out testing in squamous cell carcinoma histologies; although guidelines indicate that alterations in all genes should only be sought in patients with low or no smoking history or <50 years [11], in our population, there are four possible cases that could benefit from targeted therapy with tyrosine kinase inhibitors.

*KRAS* mutations were the most frequent alteration in our series (27.23%), a figure that aligns perfectly with prevalences reported in non-squamous NSCLC of Western populations (15–30%) [2,24] and adenocarcinomas (20–25%) [13], although slightly lower than the 36% found in some Spanish cohorts analyzed by NGS [41]. The distribution of variants within *KRAS* was also consistent, with G12C being the most common (37.40%), followed by G12V (26.84%) and G12A (6.50%), consistent with data placing G12C between 39 and 42% of total *KRAS* mutations [13,24], although slightly below the 53.6% reported in the Spanish ATLAS cohort [36]. This predominance of G12C reinforces the relevance of recently developed specific inhibitors.

Regarding histology, 81.97% of *KRAS* mutations were found in adenocarcinomas, as expected, but it is interesting to note their presence in 33.33% (17/51) of NOS, confirming their relevant role also in less differentiated tumors. Regarding squamous histology, three alterations of the G12C variant were detected in patients > 50 years, which again highlights the importance of rethinking variant testing in tumors with this histology.

The prevalence of *BRAF* mutations (5.36%) in our cohort was at the high end of the 2–8% range reported in the literature [8,9,11,41]. The proportion of the V600E variant was 33.33% (8/24), lower than the 50% usually described [8,11], indicating a significant representation of non-V600E variants in our population [66.67%], whose clinical relevance is being actively investigated.

*MET* alterations involving exon 14 skipping (*MET*ex14 skipping) were found in 8 of the 448 analyzed tumors (1.80%), a frequency below the 3–4% described in the literature [11,16,41,46,48]. *MET* amplification, on the other hand, was less frequent in our series (2/448; 0.4%) compared to reported rates of 1–6% as a primary event [13,36,49,50]. This low frequency could be real or influenced by the detection thresholds and amplification definitions used.

For *ERBB2* (*HER2*), we identified alterations in 3.57% of cases, in line with the expected 2–4% [11,20,22]. However, the variant distribution was atypical. The Y772_A775dup insertion accounted for 25% (4/16) in our cohort, even though in large cohorts of exon 20 insertions, Y772_A775dup constitutes the majority: 58% in the Chinese cohort and 41.6% in the US cohort analyzed in a multicenter study of 3000 patients. In the real-world HaploX database, the Y772_A775dup alteration was observed in 71.5% of the 284 *ERBB2* exon 20 insertions detected [51,52,53]. This could suggest a different diversity in the Canarian population. *ERBB2* amplifications (CNAs) were detected in five cases of the 448 analyzed tumors [1.12%], consistent with the 1–4% described in the consulted bibliography [11,22,36]. Regarding single-nucleotide variants, analysis of the 448 tumors showed seven alterations [43.75%].

Regarding gene fusions, our results show rates within the expected range for *ALK* (2.23% vs. 2–7%) [9,21,54], which were lower than expected for *ROS1* (0.45 vs. 1–2%) [2,11,44,54] but slightly higher than expected for *RET* (2.23% vs. 1–2%) [11,55] and *NTRK* (1.12% vs. <1%) [11]. The lower frequency of *ROS1* could be due to population factors, although the implemented RNA-based detection should maximize sensitivity [2,4,11,16,22,26,45,46,47]. The slightly elevated prevalence of *RET* and *NTRK* is an interesting finding that could indicate an enrichment in our geographic area, although absolute numbers are small.

Analysis by age groups (<50, 50–69, ≥70 years) confirms some trends described in the literature on NSCLC in young patients (AYA, ≤50 years) [19]. The most striking observation is the concentration of *ALK* fusions: four of the ten *ALK*-positive cases (40%) occurred in the <50 years group, which only represents 2.7% of the total cohort. This translates to an *ALK* prevalence of 33.33% (4/12) in this group, much higher than the 2.2% globally and in line with the 10–25% enrichment described in AYA [19]. Conversely, *KRAS* mutations were infrequent in those under 50 years (16.7%, 2/12), concentrating mostly in the 50–69 years group (66.4% of all *KRAS*, 81/122), supporting the association of *KRAS* with older ages and possibly greater cumulative tobacco exposure [19,46]. The single *EGFR* mutation detected in <50 years (8.3%) suggests a low prevalence in this group in our cohort, although the literature varies on this [19]. Our data, although limited in the youngest group, reinforce the concept of distinct molecular profiles according to age, highlighting the importance of screening for fusions in young patients. Therefore, age should not be a deterrent for testing actionable fusions, particularly *ALK*, even in the absence of other clinical risk factors.

Correlation with sex also showed patterns consistent with the literature. There was a higher frequency of *EGFR* mutations in women (39 women vs. 24 men), which was especially marked in the ≥70 years group (18 vs. 10), and there was a higher prevalence of *KRAS* (75 men vs. 47 women) and *BRAF* (16 men vs. 8 women) mutations in men [2,22,46]. These findings reaffirm the epidemiological and possibly biological differences linked to sex in NSCLC.

Analysis of smoking status in our cohort (Table 3 and Table 8) confirms several key associations and reveals interesting findings. As expected, we observed an almost absolute association of squamous cell carcinoma with smoking (99.1% of cases in smokers/former smokers), as well as the expected high prevalence of *KRAS* (89.3%) and *TP53* (81.1%) mutations in this same patient group. This agrees with the literature, which links these alterations to high tumor mutational burden (TMB) and exposure to tobacco carcinogens [3]. Specifically, our data showed that *KRAS* G12C was the most prevalent variant (45.1%), aligning with the description of G12C and G12V variants as dominant in smokers [8,11,13,36,46]. We also observed that the vast majority of *BRAF* mutations (22/24 cases, 91.7%) were associated with smoking. This fact could correlate with our high proportion of non-V600E variants (58.3%), as the literature describes that non-V600 *BRAF* mutations (Class II and III) are more common in patients with a smoking habit [11,24,46]. Regarding *MET* and *HER2*, our data (7/11 and 11/16 in smokers/former smokers, respectively) support the lack of a clear statistical association with smoking, as described in the literature [2,3,22,24,41,50].

However, the most striking finding of our cohort is the distribution of driver alterations classically associated with non-smokers (*EGFR*, *ALK*, *ROS1*, *RET*). Although the literature indicates an *EGFR* prevalence of up to three times higher in non-smokers [2,26,44], in our series, the majority of *EGFR* mutations (34/63, 54.0%) were detected in patients with a history of smoking (18 smokers, 16 former smokers) compared to only 22 cases in non-smokers. Similarly, *ALK* fusions, traditionally linked to non-smokers [11,46,54,56], were found in five out of ten cases (50%) in patients with a smoking history. Although we did not find *ROS1* or *RET* fusions in non-smokers, our numbers are too small to establish a trend. These data on *EGFR* and *ALK*, together with the 14 *EGFR* cases in squamous cell carcinomas, suggest that, in our population, smoking should not be an exclusion factor for seeking key actionable alterations. These results strongly support the implementation of universal NGS testing for all NSCLC patients, regardless of their smoking history.

Finally, the high prevalence of *TP53* alterations (30.8%, underestimated due to the panel change) agrees with its role as the most frequently altered gene in NSCLC and its frequent co-occurrence with other drivers [25,41,42,55]. Full characterization of *TP53* variants and their correlation with other genes and prognosis will be the subject of future analysis once complete data with the OPA panel is available.

One of the most relevant findings enabled by NGS through the use of panels is the identification of concomitant genomic alterations in the same tumor. The traditional paradigm considering driver mutations as mutually exclusive events has been superseded, as current evidence demonstrates that the coexistence of multiple alterations is a more frequent phenomenon than initially estimated, with profound clinical implications [42,57]. Although the prevalence of the coexistence of two or more actionable drivers is low, reported in around 1.5–1.7% of NSCLC patients [42,58], comprehensive genomic analysis reveals that up to 82.8% of tumors with a known driver harbor at least one additional pathogenic co-alteration [57]. In our series, we identified 172 cases with more than one alteration [69.6% of tumors with molecular alterations], and notably, 86 cases presented concomitant alterations among the ten main genes analyzed in this series [34.8% of tumors with molecular alterations]. This underscores the limited scope of single-gene testing.

Our data confirms *TP53* as the predominant co-driver in NSCLC, present in 29.5% of cases and frequently co-occurring with *KRAS* (5.6%) and *EGFR* (2.5%). This high prevalence is clinically critical because *TP53* co-mutations are not merely passenger events; they are established negative prognostic factors associated with reduced responsiveness to tyrosine kinase inhibitors in *EGFR*-mutant patients [59] and variable responses to immunotherapy in *KRAS*-mutant tumors [25]. By capturing these complex *TP53*-driven profiles, our study demonstrates that comprehensive genomic profiling is essential not just for diagnosis but for accurate prognostic stratification.

Finally, our study has limitations inherent to its retrospective, single-center design, which may limit the generalizability of the findings to broader populations. Furthermore, the technological transition during the study period introduces a bias in the estimation of variant frequencies. The switch from the Oncomine Focus Assay (OFA) to the Oncomine Precision Assay (OPA) means that genes such as *TP53* were not sequenced in samples from the first period (2023), leading to an underestimation of their overall prevalence in the total cohort. Conversely, genes like *ARAF*, present in OFA coverage but not in OPA, were only assessed in the first cohort.

## 5. Conclusions

Our two-year experience at HUGCDN confirms the feasibility and necessity of NGS in NSCLC. We have characterized the genomic profile of the Canarian population, observing a prevalence of *KRAS* (27.2%) and *EGFR* (14.1%) consistent with Caucasian populations, but with particularities such as an atypical distribution of *EGFR* variants (ex19del 35.6% vs. L858R 16.4%) and *BRAF* (V600E 33.3%).

Findings with direct clinical implications stand out, such as the high frequency of *ALK* fusions in young patients (33.3% in <50 years) and the surprising prevalence of drivers such as *EGFR* (54.0%) and *ALK* (60.0%) in patients with a smoking history. These data, together with the detection of complex co-mutation profiles (34.8% of cases), demonstrate the value of NGS for optimizing therapeutic management in our population, suggesting that smoking should not be an exclusionary factor for testing canonical drivers.

Comprehensive molecular diagnosis of all NSCLC via NGS, regardless of stage and histological type, is emerging as the global diagnostic standard. Continuous research into new biomarkers and targeted therapies, along with the evolution of NGS platforms (including whole exome sequencing in the near future), improved resistance detection, and disease monitoring, promise to further optimize the management of this complex disease.

## Figures and Tables

**Table 1:** Differences between OFA and OPA panels.

	OFA	OPA
**Number of DNA Genes**	35	45
**Number of Fusion Genes (RNA)**	23	20
***TP53* Detection**	No	Yes
**Usage Period**	1 March 2023–31 December 2023	1 January 2024–31 March 2025

**Table 2:** Characteristics of the study population. NOS: Not Otherwise Specified. * Includes large cell neuroendocrine carcinoma (LCNEC), adenosquamous carcinoma, and pleomorphic carcinoma.

	Subgroup	*n* (%)
**Age (years)**	<50	12 (2.7%)
	50–69	259 (58.1%)
	≥70	175 (39.2%)
	Median (Range)	67 (34–94)
**Patient Gender**	Male	286 (64.1%)
	Female	160 (35.9%)
**Histological Type**	Adenocarcinoma	267 (59.6%)
	Squamous Cell Carcinoma	119 (26.6%)
	NOS	52 (11.6%)
	Others *	10 (2.2%)
**TNM Stage (8th ed.)**	Stage I (IA, IB)	81 (18.1%)
	Stage II (IIA, IIB)	43 (9.6%)
	Stage III (IIIA, IIIB, IIIC)	79 (17.7%)
	Stage IV	226 (50.4%)
	Not Available	19 (4.2%)
**Smoking Status**	Smoker	205 (45.9%)
	Former Smoker	148 (33.2%)
	Non-smoker	40 (8.9%)
	Not Available	53 (11.9%)

**Table 3:** Distribution of histological types according to smoking status. (N/A: data not available).

	Smoker	Former Smoker	Non-Smoker	N/A
**Adenocarcinoma**	117	85	37	28
**Squamous Cell Carcinoma**	62	50	1	6
**NOS**	20	13	2	17
**Others**	7	1	-	2

**Table 4:** Number of tumors with alterations in each gene and frequency relative to total tumors.

Altered Gene	No. of Tumors	Frequency (%)
***TP53***	132	29.46%
***KRAS***	122	27.23%
***EGFR***	63	14.06%
***BRAF***	24	5.36%
***ERBB2***	16	3.57%
***MET***	11	2.46%
***ALK***	10	2.23%
***RET***	10	2.23%
***NTRK***	5	1.12%
***ROS1***	2	0.45%

**Table 5:** Distribution of genomic alterations by histological types.

	Adenocarcinoma	Squamous Cell Carcinoma	NOS	Others
***ALK***	10	-	-	-
***BRAF***	19	3	2	-
***EGFR***	46	14	3	-
***KRAS***	100	4	17	1
***ROS1***	1	-	1	-
***MET***	9	1	1	-
***RET***	6	3	-	1
***NTRK***	3	1	1	-
***ERBB2***	11	3	2	
***TP53***	71	45	14	2

**Table 6:** Distribution of alterations by tumor stage.

	IA	IB	IIA	IIB	IIIA	IIIB	IIIC	IV	N/A
***ALK***	-	-	-	-	1	1	-	8	-
***BRAF***	5	2	1	2	-	1	1	12	-
***EGFR***	4	4	1	3	3	5	1	39	3
***KRAS***	17	11	1	8	6	3	2	71	3
***ROS1***	1	-	-	-	-	-	-	-	-
***MET***	1	1	-	2	-	-	-	7	-
***RET***	1	-	-	-	-	-	-	9	-
***NTRK***	1	-	-	1	-	-	-	3	-
***ERBB2***	1	-	-	2	2	1	-	9	1
***TP53***	13	9	2	9	9	10	5	69	6

**Table 7:** Distribution of genomic alterations by sex and age group.

	Male			Female		
	<50	50–69	>70	<50	50–69	>70
***ALK***	1	4	-	3	2	-
***BRAF***	-	7	9	-	7	1
***EGFR***	-	14	10	1	20	18
***KRAS***	2	41	32	-	40	7
***ROS1***	-	1	-	-	-	1
***MET***	-	2	3	-	3	3
***RET***	-	3	3	-	4	-
***NTRK***	-	1	-	-	3	1
***ERBB2***	-	8	3	-	1	4
***TP53***	2	46	35	1	31	17

**Table 8:** Distribution of genomic alterations by smoking status. N/A: data not available.

	Smoker	Former Smoker	Non-Smoker	N/A	TOTAL
***ALK***	5	1	3	1	10
***BRAF***	12	10	2	-	24
***EGFR***	18	16	22	7	63
***KRAS***	61	48	4	9	122
***ROS1***	-	-	-	2	2
***MET***	3	4	1	3	11
***RET***	4	6	-	-	10
***NTRK***	1	1	1	2	5
***HER2***	6	5	3	2	16
***TP53***	69	38	11	14	132

**Table 9:** Distribution of genomic alterations of the six most frequent variants in *KRAS* by smoking status. N/A: data not available.

	Smoker	Former Smoker	Non-Smoker	N/A
**G12C**	27	16	1	2
**G12V**	15	14	1	3
**G12A**	5	3	-	-
**G13D**	2	4	-	-
**G12D**	1	4	1	1
**G13C**	4	1	-	1

**Table 10:** Concomitant genomic alterations matrix of the 10 main genes.

	*ALK*	*BRAF*	*EGFR*	*HER2*	*KRAS*	*MET*	*NTRK*	*RET*	*ROS1*	*TP53*
***ALK***	-	-	-	-	-	-	-	-	-	3
***BRAF***	-	-	2	-	4	-	-	-	-	7
***EGFR***	-	2	2	2	2	-	-	-	-	11
***HER2***	-	-	2	-	-	-	-	1	-	3
***KRAS***	-	4	2	-	1	-	-	2	-	25
***MET***	-	-	-	-	-	-	-	-	-	1
***NTRK***	-	-	-	-	-	-	-	-	-	1
***RET***	-	-	-	1	2	-	-	-	-	-
***ROS1***	-	-	-	-	-	-	-	-	-	-
***TP53***	3	7	11	3	25	1	1	-	-	3

## Data Availability

The data presented in this study are available upon request from the corresponding author. The data are not publicly available due to ethical and privacy restrictions regarding human genomic information.

## Supplementary Materials

The following supporting information can be downloaded at https://www.mdpi.com/article/10.3390/genes17020209/s1, Table S1: Distribution of variants in the *EGFR* gene; Table S2: Distribution of variants in the *KRAS* gene; Table S3: Distribution of variants in the *BRAF* gene; Table S4: Distribution of variants in the *ALK* gene; Table S5: Distribution of variants in the *MET* gene; Table S6: Distribution of variants in the *RET* gene; Table S7: Distribution of variants in NTRK family genes; Table S8: Distribution of variants in the *ROS1* gene; Table S9: Distribution of variants in the *ERBB2* gene.
