# Supplementary material for: Genomic Profile of Non-Small Cell Lung Cancer in a Spanish Cohort: A 2-Year Descriptive Study Using Next-Generation Sequencing
Source: Genes (Basel). 2026 Feb 9;17(2):209. doi: 10.3390/genes17020209 (PMC12940961; doi:10.3390/genes17020209)
Supplement: Supplementary file 1 [file genes-17-00209-s001.zip › genes-4059941-SI.pdf]

Alteration	Cases
**Exon 19 del**	26
**CNAs (gain)**	22
**L858R**	12
**Exon 20 ins**	4
**S768I**	2
**V774M**	2
**Indel E709-E710**	2
**L861Q**	1
**G1054W**	1
**V843L**	1

Table S1 – Distribution of variants in the *EGFR* gene.

Alteration	Cases
**G12C**	46
**G12V**	33
**G12A**	8
**G12D**	7
**G13D**	6
**G13C**	6
**Q61H**	4
**Q61L**	3
**CNAs**	2
**G12R**	2
**G12R**	1
**D30E**	1
**Q61R**	1
**A146T**	1
**G12S**	1
**V8L**	1

Table S2 – Distribution of variants in the *KRAS* gene.

Alteration	Cases
**V600E**	8
**G469A**	3
**G464V**	3
**G596R**	3
**D594N**	2
**G466R**	2
**G469R**	1
**K601E**	1
**L597V**	1

Table S3 – Distribution of variants in the *BRAF* gene.

Alteration	Cases
EML(13)::ALK(20)	3
EML(6)::ALK(20)	3
F1027L	1
M1273T	1
L1187M	1
Intronic	1

Table S4 – Distribution of variants in the *ALK* gene

Alteration	Cases
ex14 skipping (RNA)	7
D1028N (ex14 skipping)	2
CNA (gain)	2
H1112Y	1

Table S5 – Distribution of variants in the *MET* gene.

Alteration	Cases
R886Q	3
CCDC6(1)::RET(12)	1
KIF5B(15)::RET(12)	1
Imbalance	1
R886W	1
V804M	1
E768K	1
CNA (loss)	1

Table S6 – Distribution of variants in the *RET* gene.

Alteration	Cases
CNA (gain)	2
TPM3(7)::NTRK1(10)	1
TPR(21)::NTRK1(12)	1
STRN(3)-NTRK2(15)	1

Table S7 – Distribution of variants in *NTRK* family genes.

Alteration	Cases
EZR(10)::ROS1(34)	1
CD74(6)::ROS1(34)	1

Table S8 – Distribution of variants in the *ROS1* gene.

Alteration	Cases
CNA (gain)	5
Y772_A775dup	4
S310Y	2
D769H	1
R683Q	1
I370M	1
G660D	1
S310F	1

Table S9 – Distribution of variants in the *ERBB2* gene.
